# Differences in Residency Advising and Mentorship for Osteopathic and Allopathic Emergency Medicine Applicants

**DOI:** 10.7759/cureus.112951

**Published:** 2026-07-19

**Authors:** Rowan H Kelner, Megan Fix, Patrick Hughes, Robert Stephen, Brian Merritt, Julia Ruggieri, Allison M Beaulieu

**Affiliations:** 1 Emergency Medicine, The University of Utah, Salt Lake City, USA

**Keywords:** advising, education, emergency medicine, medical student, mentorship

## Abstract

Background: There has been a notable increase in osteopathic (DO) medical students applying to emergency medicine (EM) residency programs following the integration of the graduate medical education (GME) accreditation system. Despite this growth, DO students face significant challenges, including limited access to home EM residency programs and specialty-specific advising compared to their allopathic (MD) counterparts. Previous research indicates substantial dissatisfaction among DO students regarding the availability and quality of EM residency advising. Consequently, there is a critical need to identify specific advising disparities between DO and MD applicants to improve the residency application experience. The purpose of this study was to evaluate differences in residency application advising and mentorship between DO and MD medical students applying to emergency medicine.

Methods: Fourth-year medical students completing an EM sub-internship at a single Mountain West academic institution between June and December 2023 were invited to complete an anonymous 16-item survey assessing advising in three domains: EM sub-internship preparation, Electronic Residency Application Service (ERAS) application preparation, and interview preparation and communication. Demographic data included degree type (DO/MD) and gender. Categorical variables were summarized as n (%). MD and DO students were compared using chi-square or Fisher’s exact tests, as appropriate. Statistical significance was defined as p < 0.05.

Results: Twenty-nine of 41 eligible students completed the survey (71%), including 14 (48%) DO and 15 (52%) MD students; 16 (55%) were male and 13 (45%) were female. MD students were more likely than DO students to have a home EM residency program, 13 (87%) versus 4 (29%) (p = 0.003). No differences were identified in training on presentations, assessment and plans, or documentation. However, MD students were more likely to receive coaching on calling consultants/admitting services, 10 (67%) versus 1 (7%) (p = 0.002). MD students also more frequently received advising on residency program selection, 7 (47%) versus 1 (7%) (p = 0.035). Non-significant trends favored MD students for feedback on ERAS experiences, 7 (47%) versus 2 (14%) (p = 0.11), impactful experiences, 6 (40%) versus 2 (14%) (p = 0.21), mock interviews, 13 (87%) versus 7 (50%) (p = 0.050), and post-interview communication guidance, 10 (67%) versus 4 (29%) (p = 0.066). No significant differences were observed by gender.

Conclusions: This study demonstrates significant disparities between osteopathic and allopathic EM residency applicants in access to advising and mentorship, especially in home EM residency programs, communication training with consultants, and program selection guidance. The results highlight the necessity for targeted interventions and structured advising resources to support DO students, thereby promoting equitable opportunities for all applicants.

## Introduction

In recent years, emergency medicine (EM) has remained one of the most popular residency specialties among osteopathic (DO) medical students [1]. Following the implementation of the single accreditation system for graduate medical education (GME) in 2020, osteopathic representation in EM has increased steadily, reflecting both growing interest in the specialty and expanded training opportunities in the United States [2,3]. By 2025, DO seniors comprised approximately one-third of all EM applicants (1,231 of 3,753), an increase from 28% in 2024 (1,013 of 3,574), while allopathic (MD) seniors continued to represent the largest applicant group nationally [4]. As the proportion of osteopathic applicants continues to grow within an increasingly competitive residency landscape, understanding their experiences and preparation strategies has become increasingly important. Despite the expanded integration of osteopathic and allopathic training pathways, DO students may continue to face unique challenges during the residency application process, including obtaining specialty-specific advising, navigating audition rotations, and understanding application expectations [5].

Numerous online resources, such as program directories, specialty-specific advising websites, and mentorship platforms, are available to medical students pursuing EM [6]. These tools provide a wealth of general information regarding program requirements, application strategies, and interview preparation. However, there remains a paucity of data on the unique advising needs of DO students compared to their MD peers. Previous work has shown that a minority of osteopathic medical schools are affiliated with an EM residency [7] and that only 20% of DO students (compared to 70% of MD students) have EM-specific mentorship [2]. In previous surveys, a majority of DO students were dissatisfied with their advising [2].

The Council of Emergency Medicine Residency Directors (CORD) Advising Students Committee in Emergency Medicine (ASC-EM) has previously published student advising recommendations for general EM applicants in an effort to disseminate standardized information to students and potential advisors [6]. Despite also publishing specific recommendations for DO students in 2018, there still exists a large unmet need for student advising, particularly for osteopathic students [5]. This investigation is consequential because it seeks to identify DO-specific advising and mentoring needs in order to better serve this growing group of EM residency applicants. The broad dissatisfaction with mentorship reflects an unmet need [2]. Further, a closer understanding of specific osteopathic advising needs should translate into more prepared applicants.

Our study seeks to address this gap by examining the specific advising needs of DO students applying to EM.

## Materials and methods

Study design and setting

This cross-sectional survey study was approved and deemed exempt by the Institutional Review Board. Fourth-year medical students who completed an EM sub-internship at a single academic institution in the Mountain West between June 2023 and December 2023 were invited to participate. The institution is a large public, research-intensive academic medical center that serves a diverse urban, suburban, rural, and regional referral population and supports undergraduate medical education, residency training, and fellowship programs within its Department of Emergency Medicine.

Selection of participants

All students completing an EM sub-internship at the study institution, including both home and visiting students, were eligible for inclusion. Participants were invited to complete the survey after completion of the rotation and receipt of their final grade. Participation was voluntary, and responses were collected anonymously.

Measurements

A 16-item survey was developed by the study team based on key advising and mentorship topics relevant to the emergency medicine residency application process and administered using Google Forms (Google LLC, Mountain View, California). Survey questions evaluated advising and mentorship in three domains: (1) EM sub-internship clinical preparation, including expectations for clinical performance, obtaining standardized letters of evaluation, and navigating away rotations; (2) Electronic Residency Application Service (ERAS) application preparation, including application components, program selection, personal statement development, and application strategy; and (3) interview preparation and communication, including interview expectations, mock interview preparation, post-interview communication, and residency ranking considerations (Appendices). Participants also provided demographic information, including degree type (Doctor of Osteopathic Medicine (DO) or Doctor of Medicine (MD)) and gender. Receipt of advising or mentorship on specific topics was assessed using dichotomous (yes/no) response options.

Statistical analysis

Analyses were performed using Python 3.11 (Python Software Foundation, Beaverton, Oregon) with the pandas (The pandas development team, NumFOCUS, Austin, Texas) and SciPy (SciPy Community, NumFOCUS, Austin, Texas) libraries [8]. Categorical responses are summarized as n (%). MD and DO respondents were compared using Pearson’s chi-square test without continuity correction when all cells contained at least five observations and Fisher’s exact test otherwise. Odds ratios with 95% confidence intervals are reported as effect estimates. Tests were two-sided; p < 0.05 was considered significant. P-values were not adjusted for multiple comparisons.

## Results

A total of 29 students completed the survey, yielding a response rate of 29/41 (71%). Degree type and gender were distributed relatively equally among respondents (DO: 14 (48%), MD: 15 (52%); male: 16 (55%), female: 13 (45%)). Allopathic medical students were significantly more likely than osteopathic students to have a home emergency medicine residency program, 13 (87%) versus 4 (29%) (p = 0.003) (Table 1). 

**Table 1:** Respondent demographics and characteristics Data are presented as n (%). Test statistics and p-values compare MD and DO respondents (chi-square test where a test statistic is shown; Fisher’s exact test, which yields no test statistic, shown as —); degree is the grouping variable and is not tested. MD: Doctor of Medicine; DO: Doctor of Osteopathic Medicine; EM: emergency medicine; Sub-I: sub-internship. Credits: Rowan Kelner and Allison Beaulieu. Questionnaire: authors’ original creation.

Characteristics	Overall (N = 29), n (%)	DO (N = 14), n (%)	MD (N = 15), n (%)	Test statistic	p-value
Degree					
DO	14 (48%)	—	—		
MD	15 (52%)	—	—		
Gender				χ² = 0.91 (df = 1)	0.34
Female	13 (45%)	5 (36%)	8 (53%)		
Male	16 (55%)	9 (64%)	7 (47%)		
Home clerkship (with EM residency program)				—	0.003
Yes	17 (59%)	4 (29%)	13 (87%)		
No	12 (41%)	10 (71%)	2 (13%)		
EM rotation prior to Sub-I				—	>0.99
Yes	28 (97%)	14 (100%)	14 (93%)		
No	1 (3%)	0 (0%)	1 (7%)		

No significant differences were identified between MD and DO students regarding preparation for emergency medicine sub-internships, including training in patient presentations, assessment and plan development, and note writing. However, MD students were significantly more likely to report receiving instruction on calling consultants and admitting services, 10 (67%) versus 1 (7%) (p = 0.002) (Table 2). 

**Table 2:** Emergency medicine sub-internship clinical preparation Data are n (%). p-values compare MD and DO respondents. Chi-square (χ²) values are shown where applicable; Fisher’s exact test yields no test statistic (—). OR: odds ratio (odds of an affirmative response in MD versus DO); CI: confidence interval; MD: Doctor of Medicine; DO: Doctor of Osteopathic Medicine; EM: emergency medicine; ERAS: Electronic Residency Application Service. Credits: Rowan Kelner and Allison Beaulieu. Questionnaire: authors’ original creation.

Survey item	DO (N = 14), n (%)	MD (N = 15), n (%)	OR (95% CI)	Test statistic	p-value
On-shift expectations explained	13 (93%)	13 (87%)	0.51 (0.01–10.99)	—	>0.99
EM presentation coaching	9 (64%)	9 (60%)	0.84 (0.14–4.77)	χ² = 0.06 (df = 1)	0.81
Assessment & plan coaching	8 (57%)	9 (60%)	1.12 (0.20–6.28)	χ² = 0.02 (df = 1)	0.88
Documentation coaching	7 (50%)	6 (40%)	0.68 (0.12–3.65)	χ² = 0.29 (df = 1)	0.59
Consultant-calling coaching	1 (7%)	10 (67%)	22.69 (2.27–1212)	—	0.002

With respect to the residency application process, MD students were significantly more likely to receive advising on residency program selection compared with DO students, 7 (47%) versus 1 (7%), p = 0.035. Although these differences did not reach statistical significance, MD students more frequently reported receiving guidance on writing the 10 ERAS experiences entries, 7 (47%) versus 2 (14%), p = 0.11, and the impactful experience statement, 6 (40%) versus 2 (14%), p = 0.21 (Table 3). 

**Table 3:** ERAS application advising Data are n (%). p-values compare MD and DO respondents. Fisher’s exact test yields no test statistic (—). OR: odds ratio (odds of an affirmative response in MD versus DO); CI: confidence interval; MD: Doctor of Medicine; DO: Doctor of Osteopathic Medicine; EM: emergency medicine; ERAS: Electronic Residency Application Service. Credits: Rowan Kelner and Allison Beaulieu. Questionnaire: authors’ original creation.

Survey item	DO (N = 14), n (%)	MD (N = 15), n (%)	OR (95% CI)	Test statistic	p-value
ERAS experience section feedback	2 (14%)	7 (47%)	4.95 (0.70–60.88)	—	0.11
Impactful experience feedback	2 (14%)	6 (40%)	3.81 (0.52–47.34)	—	0.21
Residency application advice	1 (7%)	7 (47%)	10.47 (1.04–551)	—	0.035

No statistically significant differences were observed between MD and DO students in interview preparation or post-interview communication. Nevertheless, MD students more commonly reported receiving advising on interview preparation, 13 (87%) versus 7 (50%), p = 0.050, and post-interview communication, 10 (67%) versus 4 (29%), p = 0.066 (Table 4).

**Table 4:** Interview preparation and communication Data are n (%). p-values compare MD and DO respondents. Fisher’s exact test yields no test statistic (—). OR: odds ratio (odds of an affirmative response in MD versus DO); CI: confidence interval; MD: Doctor of Medicine; DO: Doctor of Osteopathic Medicine; EM: emergency medicine; ERAS: Electronic Residency Application Service. Credits: Rowan Kelner and Allison Beaulieu. Questionnaire: authors’ original creation.

Survey item	DO (N = 14), n (%)	MD (N = 15), n (%)	OR (95% CI)	Test statistic	p-value
Mock interview opportunity	7 (50%)	13 (87%)	6.06 (0.85–75.40)	—	0.050
Post-interview communication guidance	4 (29%)	10 (67%)	4.70 (0.82–32.78)	—	0.066

No significant differences were identified between male and female respondents for any survey item.

## Discussion

The findings of this study highlight significant differences in advising between DO and MD medical students applying to EM residency programs, underscoring critical areas for improvement in residency application advising and mentoring for osteopathic students. While both groups demonstrated comparable preparation for specific aspects of their EM sub-internships, such as presentations, assessments, and note writing, the disparity in advising resources and training became apparent in other critical areas of the residency application process. Notably, MD students were more likely to benefit from home EM residency programs, formal training on calling consultants and admitting services, and advising on residency program selection. These gaps reveal potential barriers for DO students and suggest that their advising needs are not being fully addressed.

A particularly striking finding was the disparity in access to a home EM residency program, defined as an emergency medicine residency affiliated with a student's medical school or primary clinical training institution, with 87% of MD students reporting access compared to only 31% of DO students. Home programs are critical for providing specialty-specific mentorship, clinical experience, opportunities to obtain Standardized Letters of Evaluation (SLOEs), and guidance throughout the residency application process, given their proximity to GME training structures. Prior emergency medicine literature has demonstrated that osteopathic students often have less affiliation with EM residency programs, reduced access to EM-specific mentorship, and fewer institution-based advising resources than their allopathic peers [2,5]. The absence of a home program likely places many DO students at a disadvantage by limiting exposure to the specialty, networking opportunities, and access to faculty experienced in advising residency applicants [9,10]. This finding underscores the need for national initiatives and cross-institutional partnerships to ensure equitable access to advising and mentorship for all EM-bound students, regardless of institutional affiliation [10].

Another key finding was the limited training received by DO students in calling consultants and admitting services, a critical skill in EM practice. MD students were significantly more likely to receive this training (67% vs. 8%), reflecting a potential gap in the clinical preparation of DO students. The Clerkship Directors in Emergency Medicine have developed a standardized curriculum available to all students and educators; however, there is no osteopathic-specific content that may better prepare DO students for both their rotations and future residency training.

Advising on residency program selection was another area of significant disparity, with MD students more likely to receive tailored guidance (47% vs. 8%). Effective advising on program choice is essential for aligning applicants' goals with program expectations and improving match outcomes. The CORD has attempted to address these concerns by creating a virtual mentoring option for students lacking adequate advising [11]. However, this finding emphasizes the need for more targeted efforts to ensure DO and orphan students receive comparable support, potentially through proactive mentorship programs or expanded virtual advising initiatives that connect applicants with experienced advisors.

While other advising domains, including ERAS application preparation, interview preparation, and post-interview communication, did not demonstrate statistically significant differences, the observed trends suggest that MD students may receive more robust advising overall. Importantly, advising for MD students was not universally available. Across many advising domains, only 40%-60% of MD students reported receiving advising or mentorship. This finding highlights the need for improved advising resources for all EM applicants, not solely DO students.

Several factors may contribute to these observed trends. Access to specialty-specific advising often depends on the availability of EM faculty mentors, residency-affiliated educators, and formal departmental advising structures. Students training at institutions without an affiliated EM residency program may have fewer opportunities for mentorship and career guidance and may therefore rely more heavily on away rotations or external professional networks. Additionally, the Mountain West region encompasses a large geographic area with relatively few academic medical centers, potentially creating further challenges in accessing EM mentors and residency-related resources. However, the widespread advising gaps observed among both MD and DO students suggest that limitations in faculty availability, mentorship capacity, and formal advising infrastructure may affect EM applicants regardless of degree pathway.

Previous research has demonstrated that advising regarding residency competitiveness and program selection is inadequate across multiple specialties [12]. Advising deficiencies are therefore not unique to emergency medicine but represent a broader challenge within undergraduate medical education. Furthermore, mentorship and advising concerns frequently extend into residency training and remain an active area of educational research [13,14].

Future directions include repeating ASC-EM and CORD advising and mentorship surveys to evaluate progress and identify persistent needs. Advising resources will also need to evolve alongside the implementation of ResidencyCAS and should include targeted support for DO, international medical graduates (IMGs), and orphan applicants who lack access to a home EM residency program [15]. Given that these groups often experience less structured advising and mentorship, we encourage EM sub-internship sites to proactively provide targeted mentorship and support.

This study has several limitations. The small sample size and single-center design limit the generalizability of the findings. Additionally, the study is subject to response and recall bias, as participation was voluntary and advising experiences were assessed through retrospective self-report. Nonetheless, these results provide valuable insight into advising disparities among EM applicants and identify actionable opportunities to improve the residency application process.

## Conclusions

In conclusion, this study identified disparities in emergency medicine advising between MD and DO students across multiple stages of the residency application process. Although both groups reported similar preparation in several core clinical competencies, MD students were more likely to receive guidance on consultant communication and residency program selection, with additional trends favoring MD students in ERAS application support and interview preparation. These findings suggest that differences in access to advising resources, mentorship, and home residency programs may contribute to unequal preparation for the residency application process. Developing a more structured, equitable, and inclusive advising framework, particularly for students without access to a home EM residency program, may help reduce these gaps and ensure that all applicants receive the support needed to navigate the increasingly competitive residency match process. By investing in tailored mentorship and advising initiatives, the EM community can better promote applicant success, strengthen diversity within the specialty, and cultivate a well-prepared future workforce.

## Appendices

**Table 5:** MS4 EM sub-internship mentorship targeted needs assessment This questionnaire is the authors’ original creation, developed for a curriculum targeted needs assessment. The instrument was administered electronically (Google Forms) and is reproduced here in its English version. Questions 1-14 were analyzed in Tables 1-4; Questions 15-16 were collected for program-planning purposes and were not included in the comparative analyses. EM: emergency medicine. Credits: Rowan Kelner and Allison Beaulieu.

#	Question	Response Options
1	What degree are you working towards?	☐ MD ☐ DO
2	What is your preferred gender?	☐ Male ☐ Female ☐ Non-binary ☐ Prefer not to say
3	Does your program have a home clerkship with an associated EM residency program?	☐ Yes ☐ No
4	Did you complete an EM rotation prior to starting your Sub-I? (Examples: 3rd-year elective or 4th-year required rotation)	☐ Yes ☐ No
5	Prior to your first EM Sub-I, were on-shift expectations and etiquette explained?	☐ Yes ☐ No
6	Prior to your first EM Sub-I, did you get coaching on how to do an EM presentation?	☐ Yes ☐ No
7	Prior to your first EM Sub-I, did you get coaching on how to do an EM assessment and plan?	☐ Yes ☐ No
8	Prior to your first EM Sub-I, did you get coaching on how to do EM documentation?	☐ Yes ☐ No
9	Prior to your first EM Sub-I, did you get coaching on how to call a consultant?	☐ Yes ☐ No
10	Did you get personalized feedback on how to fill out your ERAS experience section?	☐ Yes ☐ No
11	Did you get personalized feedback on how to write your impactful experience?	☐ Yes ☐ No
12	Did you have personalized feedback on which residencies to apply to?	☐ Yes ☐ No
13	Did you have the opportunity to perform a mock interview with faculty?	☐ Yes ☐ No
14	Were you provided with guidelines on and help with post-interview communication?	☐ Yes ☐ No
15	Select the Top 5 categories most important to you to receive personalized feedback and advising (check all that apply):	☐ EM Presentation ☐ EM Documentation ☐ EM Assessment and Plan ☐ EM Consultant ☐ ERAS Personal Statement ☐ ERAS Top 10 Experiences ☐ ERAS Impactful Experience ☐ Interview Preparation with Mock Interview ☐ Advising on Choosing Residency Programs ☐ Advising on Communicating with Programs
16	What part of the application cycle (from starting your Sub-I to interviewing) did you struggle with the most?	Free response
